# Natural history and clinical significance of MRI-detected bone marrow lesions at the knee: a prospective study in community dwelling older adults

**DOI:** 10.1186/ar3210

**Published:** 2010-12-29

**Authors:** Dawn Dore, Stephen Quinn, Changhai Ding, Tania Winzenberg, Guangju Zhai, Flavia Cicuttini, Graeme Jones

**Affiliations:** 1 Menzies Research Institute Tasmania, University of Tasmania, Private Bag 23, Hobart, 7000, Australia; 2 Department of Epidemiology and Preventive Medicine, Monash University, 89 Commercial Road, Melbourne, 3004, Australia; 3 Department of Twin Research and Genetic Epidemiology, King's College London, St Thomas' Hospital, Westminster Bridge Road, London, SE1 7EH, UK

## Abstract

**Introduction:**

There are conflicting data on the natural history and clinical significance of bone marrow lesions (BMLs). The aims of this study were to describe the natural history of MRI-detected BMLs at the knee using a quantitative measure and examine the association of BMLs with pain, function and stiffness scores, and total knee replacement (TKR) surgery.

**Methods:**

A total of 395 older males and females were randomly selected from the general population (mean age 63 years, range 52 to 79) and measured at baseline and approximately 2.7 years later. BMLs were determined using T2-weighted fat saturation MRI by measuring the maximum area of the lesion. Reproducibility was excellent (intraclass correlation coefficient (ICC): 0.97). Pain, function, and stiffness were assessed by Western Ontario and McMaster Universities Osteoarthritis (WOMAC) scores. X-ray was used to assess radiographic osteoarthritis (ROA) at baseline.

**Results:**

At baseline, 43% (*n *= 168/395) had a BML. Of these 25% decreased in size and 24% increased. Of the remaining sample (*n *= 227), 7% developed a new BML. In a multivariable model, a change in BML size was associated with a change in pain and function scores (β = 1.13 to 2.55 per 1 SD increase, all *P *< 0.05), only in those participants without ROA. Lastly, baseline BML severity predicted TKR surgery (odds ratio (OR) 2.10/unit, *P *= 0.019).

**Conclusions:**

In a population based sample, BMLs (assessed by measuring maximal area) were not static, with similar proportions both worsening and improving. A change in BML size was associated with changes in pain in those without established ROA. This finding suggests that fluctuating knee pain may be attributable to BMLs in those participants with early stage disease. Baseline BMLs also predicted TKR surgery. These findings suggest therapeutic interventions aimed at altering the natural history of BMLs should be considered.

## Introduction

Osteoarthritis (OA) is a multifactorial disease of the joints characterized by gradual loss of articular cartilage. There is strong evidence that bone plays an important role in the pathogenesis of OA and it has been suggested that bone changes may precede cartilage damage [1]. Recently we have shown that elevated tibial bone area and subchondral bone mineral density (BMD) predicted cartilage defect increases [2]. Additionally, tibial bone area predicted cartilage volume loss. Bone marrow lesions (BMLs) have also been recognized as an important feature of knee OA [3,4]. They are associated with structural changes in the knee, including joint space loss on radiographs [4], cartilage defect progression [5] and cartilage loss on MR images [5-7]. BML histology is heterogeneous and includes a mix of pathological changes. Zanetti *et al. *found that BMLs in the knee in subjects with severe OA undergoing total knee replacement consisted of several abnormalities including bone marrow necrosis, abnormal trabeculae, bone marrow fibrosis, bone marrow bleeding, and bone marrow oedema [8]. BMLs have also been described in other rheumatic conditions such as rheumatoid arthritis (RA) [9], osteonecrosis [10], ankylosing spondylitis [11], and transient osteoporosis of the hip [12] and are often referred to as bone marrow oedema (BME). In RA, it is suggested that BME represents cellular infiltrate within the subchondral bone [9] and is associated with painful and aggressive disease [13]. Although BMLs in OA and BME in RA appear similar on MR images, it is unclear whether they are under the same pathological processes.

There are conflicting data on the natural history of BMLs in knee OA. Most studies have focused on symptomatic OA populations. One study reported that <1% of patients showed a BML decrease over 30 months [6], while, in contrast, another study found that 20% of BMLs decreased over two years [14]. In subjects with prevalent knee OA or at risk for OA, Roemer *et al. *found that the majority (50%) of pre-existing BMLs decreased in size after 30 months follow-up [15]. The reasons behind these variations are unclear.

A number of studies have linked BMLs with knee pain [3,16,17] although other studies have failed to demonstrate such a relationship [14,18,19]. In pain-free populations, incident BMLs [17] and increases in BMLs [16] have been shown to be associated with development of knee pain. However, other studies in mostly OA subjects have reported no association between changes in BMLs and Western Ontario and McMaster Universities Osteoarthritis (WOMAC) index pain scores at baseline [19], WOMAC scores after two years [14], or changes in WOMAC scores [18]. Importantly, it remains unknown whether reduction or resolution of BMLs is associated with improved knee pain. Furthermore, patients with OA experience stiffness and limited function; however, there are little data on the association between function, stiffness and BMLs.

Another important clinical outcome in knee OA is joint replacement surgery. It is well-established that radiographic severity and pain are strong predictors of joint replacement surgery [20,21]; however, there have been limited prospective studies examining structural factors and knee replacement surgery. In subjects with symptomatic knee OA, ultrasound detected effusion [22], articular cartilage defects [23], rate of tibial cartilage loss and tibial bone size [24] predicted knee joint replacement. A recent study by Tanamas *et al. *showed that the severity of BMLs was positively associated with the risk of knee joint replacement in subjects with well-established OA [25]. It is unknown whether BMLs in a community-based sample also predict knee joint replacement.

The conflicting data on the natural history and clinical significance of BMLs may be due to studies grading BMLs semi-quantitatively, based on the extent of regional involvement. A truly quantitative measure of BML size may give more insight into actual changes over time. Therefore, this study aimed to: 1) describe the natural history of BMLs in a population based sample using a quantitative measure; and 2) examine the clinical correlates of BMLs, including pain, function, and stiffness scores and total knee replacement surgery.

## Materials and methods

### Subjects

This study was conducted as part of the Tasmanian Older Adult Cohort (TASOAC) study, a prospective, population-based study that was initiated in 2002 aiming to identify the environmental, genetic, and biochemical factors associated with the development and progression of OA at multiple sites (hand, knee, hip, and spine). Subjects between the ages of 50 and 80 years where randomly selected from the electoral roll in Southern Tasmania (population 229,000), with an equal number of men and women. The overall response rate was 57%. The study included a baseline examination and follow-up examinations at approximately 2.7 and 5 years. The research conducted in this manuscript is in compliance with the Helsinki Declaration and was approved by the Southern Tasmanian Health and Medical Human Research Ethics Committee. Written informed consent was obtained from all participants.

### Anthropometrics

Weight was measured to the nearest 0.1 kg (with shoes, socks, and bulky clothing removed) using a single pair of electronic scales (Seca Delta Model 707, Bradford, MA, USA). Height was measured to the nearest 0.1 cm (with shoes and socks removed) using a stadiometer. Body mass index (BMI) was calculated (kg/m^2^).

### Magnetic Resonance Imaging

An MRI of the right knee was acquired with a 1.5T whole-body magnetic resonance unit (Picker, Cleveland, OH, USA) using a commercial transmit-receive extremity coil at baseline and at the first follow-up (range 2.0 to 4.7 years, median 2.7 years). Image sequence included the following: (1) a T1-weighted fat saturation three-dimensional (3-D) gradient recall acquisition in the steady state, flip angle 30°, repetition time 31 ms, echo time 6.71 ms, field of view 16 cm, 60 partitions, 512 × 512-pixel matrix, acquisition time 5 minutes 58 seconds, one acquisition; sagittal images were obtained at a slice thickness of 1.5 mm without a interslice gap; (2) a T2-weighted fat saturation 3-D fast spin echo, flip angle 90°, repetition time 3,067 ms, echo time 112 ms, field of view 16 cm, 15 partitions, 228 × 256-pixel matrix; sagittal images were obtained at a slice thickness of 4 mm with a interslice gap of 0.5 to 1.0 mm.

**Figure 1:**
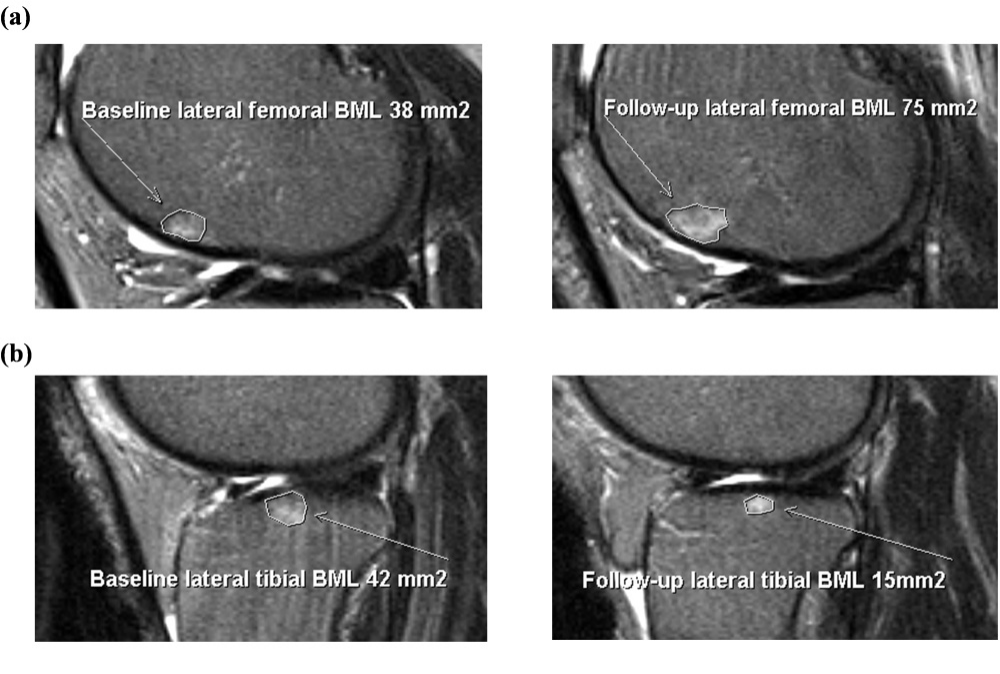
**Change in BML size**. **(a) **BML increase from baseline to first follow-up. **(b) **BML decrease from baseline to first follow-up.

Subchondral BMLs were assessed on T2-weighted MR images using Osiris software (University of Geneva, Geneva, Switzerland) and were defined as areas of increased signal adjacent to the subcortical bone at the medial tibial, medial femoral, lateral tibial, and lateral femoral sites. One trained observer scored the BMLs by measuring the maximum area of the lesion at baseline and at the first follow-up. The observer manually selected the MRI slice with the greatest BML size. The BML with the highest score was used if more than one lesion was present at the same site. The maximum area was measured in mm^2 ^using software cursors. Baseline and follow-up MRIs were read paired with the chronological order known to the observer and the observer blinded to clinical status. Intraobserver repeatability was assessed in 40 subjects with at least a two-week interval between the readings. The intraclass correlation coefficient (ICC) was 0.97. At baseline and the first follow-up, participants were given a BML score (mm^2^) for each of the four sites (medial tibial, medial femoral, lateral tibial, and lateral femoral sites) as well as a total BML score, which was the sum of the scores at each site. Figure 1a &1b illustrates a change in BML size from baseline to follow-up.

Bone marrow lesions were also assessed at baseline on an ordinal scale by a different observer as we have previously reported [26]. Each BML was scored on the basis of lesion size (for example, a lesion was scored as grade 1 if it was only present on one slice, grade 2 if present on two consecutive slices, or grade 3 if present on three or more consecutive slices). The BML with the highest score was used if more than one lesion was present at the same site. Intraobserver repeatability was assessed in 50 subjects with at least a one-week interval between the two readings with ICCs ranging from 0.89 to 1.00. The BML score was summed for all four sites to produce a total BML score (ranging from 0 to 12).

### WOMAC scores

Knee pain, function, and stiffness were assessed by self-administered questionnaire (WOMAC) [27], at baseline and the first follow-up. WOMAC uses a 10-point scale from 0 (no pain, stiffness, or function deficit) to 9 (most severe pain, stiffness or severe function problems). Knee pain, function, and stiffness assessments consisted of 5, 17, and 2 questions each; therefore, the range for each of these is from 0 to 45, 0 to 153, and 0 to 18, respectively.

In further analysis, knee pain was assessed using the five sub-scales, which included knee pain while walking on a flat surface, going up and down stairs, at night while in bed, sitting or lying, and standing upright. These ranged from 0 to 9.

Subjects also completed a questionnaire on medication use at baseline and the first follow-up.

### Knee replacement surgery

At the two follow-up visits, participants were asked whether they had undergone a total knee replacement since their first visit. Although MRI scans were taken of the right knee only at baseline, replacement surgery data were collected for both knees.

### Additional available baseline data

A standing anteroposterior semiflexed view of the right knee with 15° of fixed knee flexion was performed and scored individually for osteophytes and joint space narrowing (JSN) on a scale of 0 to 3 (0 = normal and 3 = severe) according to the Altman atlas [28] as previously described [29]. The presence of radiographic OA (ROA) was defined as any score ≥1 for JSN or osteophytes.

Leg strength was measured by a dynamometer (TTM Muscular Meter; Gloria, Tokyo, Japan) with both legs involved simultaneously. The muscles measured with this technique are predominantly quadriceps and hip flexors. Subjects were instructed in the technique before testing. Each subject had two attempts, and an average of the two was taken. The repeatability estimate was 0.91 (Cronbach's α).

The Assessment of Quality of Life (AQoL) instrument was used to measure health-related quality of life. The AQoL is a valid [30] measure of quality of life, with reliability in a population-based study of 0.81 (Cronbach's α) [31]. The total AQoL score ranged from 0 (perfect health) to 45 (worst possible health state).

Cartilage defects were assessed by a trained observer on T1-weighted MR images (score range, 0 to 4) at the tibial and femoral sites, medially and laterally, as previously described [32] as follows: grade 0 = normal cartilage; grade 1 = focal blistering and intracartilaginous low-signal intensity area with an intact surface and base; grade 2 = irregularities on the surface or base and loss of thickness <50%; grade 3 = deep ulceration with loss of thickness >50%; and grade 4 = full-thickness chondral wear with exposure of subchondral bone. A cartilage defect had to be present on at least two consecutive slices. The cartilage was considered to be normal if the band of intermediate signal intensity had a uniform thickness. If >1 defect was present on the same site the highest score was used. Intraobserver repeatability was assessed in 50 subjects with at least one week between the two measurements with ICCs ranging from 0.80 to 0.95.

Knee tibial plateau bone area was measured and defined as the cross-sectional surface area of the tibial plateau, as previously described [33-35]. The coefficient of variation (CV) in our hands for this method of measurement ranged from 2.2 to 2.6% [34].

### Statistical analysis

In order to examine the natural history of BMLs a significant change in BML size was defined as any change above (increase) or below (decrease) the least significant criterion (LSC) [36], which takes into account measurement error and the correlation between the BML measurements at baseline and follow-up. The formula was as follows:

$$LSC=1.96\times \sigma \sqrt{2(1-\rho )}$$

where *σ *is the standard error of the mean and *ρ *is the serial correlation. LSC was calculated to be 25 mm^2 ^(where *σ *= 11.67 and *ρ *= 0.3810). Therefore an increase in BML size was any change above 25 mm^2^, which included new or progressing BMLs. A decrease in BML size was any decrease greater than 25 mm^2^, which included resolved or regressing BMLs.

Logistic regression analysis was used to examine the association between baseline BMLs (absent versus present) and increases in BMLs (no increase or incident BML versus increase or incident BML) and demographic factors such as age, sex, and BMI.

Mixed effects models were used to account for the correlated readings within an individual and examine the association between changes in WOMAC scores (pain, function, and stiffness) and continuous changes in BML size. Standard diagnostic checks of model adequacy and unusual observations were performed and revealed that some of the models were heteroscedastic. This is due to the fact that much of the data is clumped at zero because BMLs were measured at four separate sites and the majority of participants who had a BML present had it at only one of the four sites. To our knowledge there is no commercially available software to deal with data of this sort in longitudinal analysis. As a result we have performed two separate analyses examining, 1) BML size change at all four sites (medial tibial, medial femoral, lateral tibial, and lateral femoral); and 2) total BML size change (all four sites combined). This was done in order to check the consistency of our results. We also stratified the analysis by presence or absence of ROA, as the results were quite different for each sub-group.

Over the course of the study period (five years), there were 12 knee replacements; therefore, we were only able to perform an exploratory analysis between BMLs and knee replacement surgery. Logistic regression and exact logistic regression modelling were used to examine whether baseline BMLs measured using the ordinal scale predicted knee replacement surgery after adjustment for potential confounders.

All statistical analyses were performed on Intercooled Stata 10.0 for windows (StataCorp, College Station, TX, USA).

## Results

### Characteristics of the study subjects

A total of 1,099 subjects (51% female) aged between 51 and 81 (mean 63 years) participated in the TASOAC study. The current study consists of a sample of 395 participants who had MRI measures at baseline and the first follow-up. MRI scans were discontinued after this sample due to decommissioning of the MRI scanner. Additional data on knee replacement surgery were available on these subjects at the second follow-up. At baseline, there were no significant differences in demographics, ROA, WOMAC function or stiffness scores, AQoL scores, or leg strength between the rest of the cohort (*n *= 704) and the subjects included in the current study (*n *= 395). There was a small difference in the WOMAC pain scores between the subjects in the current study [mean pain score 3.2 (standard deviation (SD) 6.3) compared with the rest of the cohort [mean pain score 4.1 (SD 6.4); *P *= 0.03 for difference). The characteristics of the study population are presented in Table 1.

**Table 1:** Characteristics of participants at baseline (*n *= 395)

	Value*
Age (year)	63.2 (7.2)
Male sex (%)	49
Height (cm)	167.2 (8.8)
Weight (kg)	77.3 (14.2)
BMI (kg/m^2^)	27.6 (4.5)
ROA present (%)	58
BML present (%)	43
Mean BML size (mm^2^)	72.7 (74.6)
WOMAC	
Pain (0 to 45)	3.2 (6.3)
Function deficit (0 to 153)	10.4 (21.9)
Stiffness (0 to 18)	1.5 (2.9)
Leg strength (kg)	92.3 (47.5)
AQoL (0 to 29)	7.0 (5.0)

*Mean (SD) except for percentages. AQoL, Assessment of Quality of Life; BMI, body mass index; BML, bone marrow lesion; ROA, radiographic osteoarthritis; WOMAC, Western Ontario and McMaster Universities Osteoarthritis Index.

### Natural history and demographic factors

At baseline, 43% of participants (*n *= 168/395) had one or more BML present at the medial tibial, medial femoral, lateral tibial, and/or lateral femoral site. A total of 114 subjects had a BML at one site only, 43 had a BML at two sites, 10 had a BML at three sites, and 1 had a BML at all four sites. Therefore, at all four sites combined, there were 234 total BMLs present at baseline.

The overall prevalence in those with (43%) and without ROA (41%) was similar; however, those with ROA had more total BMLs present (144) compared to those without ROA (80). In those with ROA, 58 subjects had a BML at one site only, 26 had a BML at two sites, 10 had a BML at three sites, and 1 had a BML at all four sites. Those without ROA had BMLs present at one or two sites only; 50 had a BML at one site and 15 had a BML at two sites.

Table 2 describes the association between baseline BMLs and increasing BMLs with baseline demographic factors. Those who had a BML present at baseline had a higher BMI and were more likely to be male. Males were also more likely to have a BML increase. Age or BMI did not predict BML increases.

**Table 2:** Relationship between baseline BMLs and increasing BMLs with baseline demographic factors*

	Univariate OR (95% CI)	*P*	Multivariable OR (95% CI)†	*P*
Absence/Presence of BML at baseline				
Age	1.00 (0.82, 1.22)	0.992	0.99 (0.80, 1.21)	0.905
Male sex	**1.61 (1.08, 2.41)**	**0.020**	**1.70 (1.13, 2.56)**	**0.011**
BMI	**1.31 (1.07, 1.61)**	**0.009**	**1.34 (1.09, 1.65)**	**0.005**
BML increase at any site**				
Age	1.10 (0.86, 1.40)	0.463	1.08 (0.84, 1.39)	0.529
Male sex	**1.74 (1.05, 2.86)**	**0.030**	**1.78 (1.07, 2.95)**	**0.026**
BMI	1.19 (0.94, 1.51)	0.146	1.23 (0.97, 1.58)	0.093

*Values are per one standard deviation increase in respective variable (except sex).

OR, odds ratio; 95% CI, 95% confidence interval; BMI, body mass index; BMLs, bone marrow lesions

**No increase or incident BML versus an increase or incident BML at any site (medial tibial, medial femoral, lateral tibial, and/or lateral femoral)

†Adjusted for sex and BMI in the age model, age and BMI in the sex model, or age and sex in the BMI model.

Boldface denotes statistically significant result.

**Figure 2:**
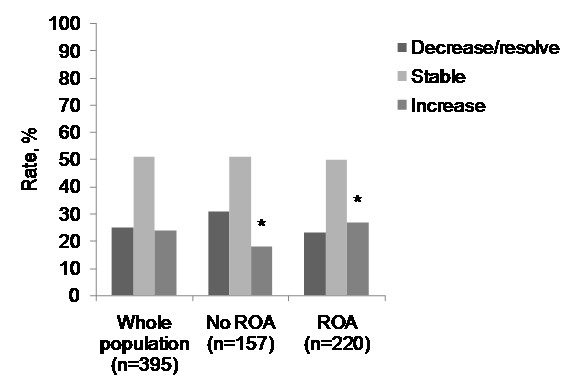
**Natural history of BMLs**. *Those with ROA had higher odds of a BML increasing compared to those without ROA (OR 2.2, *P *= 0.017).

Figure 2 describes the natural history of BMLs in the whole population and split by ROA. About half the lesions present at baseline remained stable, with similar proportions both worsening and improving. Those with ROA had higher odds of a BML increasing compared to those without ROA (odds ratio (OR) 2.2, *P *= 0.017). This was the only significant difference in the natural history between those with and without ROA.

Of those that did not have a BML at baseline (*n *= 227), 7% developed one or more BMLs from baseline to follow up. Incidence was also similar for those with (7.2%) and without ROA (6.5%).

### WOMAC scores and BMLs

Table 3 describes the association between changes in WOMAC scores and changes in BML size, stratified by ROA. A change in knee pain and function was associated with a change in BML size at all four sites, but only in those participants without ROA. These results were also consistent when using change in total BML size (all four sites combined) as the independent factor. Importantly the association between change in function and change in BML size disappeared after further adjustment for change in pain (β = 0.10 to 0.23, *P *> 0.05), demonstrating that the association between changes in function and changes in BML size is mediated by changes in pain. In those without ROA, a one SD increase in total BML size led to a 1.13-unit increase in pain (*P *= 0.009). Similarly, a one SD decrease in total BML size led to a 1.13 decrease in pain (*P *= 0.009), in those without ROA. There were no associations between changes in pain, function, or stiffness and changes in BML size (at all four sites or total BML size) in those with ROA.

**Table 3:** Relationship between changes in WOMAC scores and changes in BML size*

	BML size change at all four sites**				Total BML size change			
		
	Univariate (95% CI)	*P*	Multivariable (95% CI)†	*P*	Univariate (95% CI)	*P*	Multivariable (95% CI)†	*P*
*No ROA*								
Pain change	**0.57 (0.15, 0.99)**	**0.008**	**0.56 (0.19, 0.92)**	**0.003**	**1.06 (0.10, 2.03)**	**0.031**	**1.13 (0.28, 1.98)**	**0.009**
Function change	1.20 (-0.08, 2.47)	0.067	**1.25 (0.22, 2.28)**	**0.017**	2.23 (-0.71, 5.17)	0.136	**2.55 (0.14, 4.95)**	**0.038**
Stiffness change	-0.01 (-0.20, 0.18)	0.925	0.04 (-0.13, 0.20)	0.664	-0.02 (-0.46, 0.43)	0.947	0.09 (-0.29, 0.48)	0.641
								
*ROA Present*								
Pain change	0.07 (-0.29, 0.43)	0.715	0.03 (-0.28, 0.35)	0.844	0.11 (-0.54, 0.77)	0.733	0.06 (-0.53, 0.64)	0.848
Function change	0.16 (-0.77, 1.08)	0.740	0.13 (-0.62, 0.89)	0.729	0.26 (-1.41, 1.94)	0.756	0.23 (-1.17, 1.63)	0.750
Stiffness change	0.03 (-0.13, 0.19)	0.723	0.03 (-0.11, 0.17)	0.655	0.04 (-0.25, 0.34)	0.772	0.05 (-0.21, 0.30)	0.721

*Values are the change in pain, function, or stiffness score per one standard deviation change in BML size (mm^2^), stratified by ROA.

BML, bone marrow lesion; WOMAC, Western Ontario and McMaster Universities Osteoarthritis Index; ROA, radiographic osteoarthritis.

β, beta coefficients; 95% CI, 95% confidence interval.

**Medial tibial, medial femoral, lateral tibial, and lateral femoral.

†Adjusted for age, sex, body mass index, leg strength, quality of life, and baseline pain, function, or stiffness score depending on the model.

Boldface denotes statistically significant result

Table 4 describes the association between changes in the five WOMAC pain sub-scales and changes in BML size, stratified by ROA. Changes in knee pain when walking on a flat surface, going up and down stairs, and at night while in bed was associated with changes in BML size at all four sites, again only in those participants without ROA. These results were also consistent when using change in total BML size (all four sites combined) as the independent factor. There were no associations between changes in any of the five WOMAC pain sub-scales and changes in BML size (at all four sites or total BML size) in those with ROA.

**Table 4:** Relationship between changes in the five WOMAC pain sub-scales and changes in BML size*

	BML size change at all 4 sites **				Total BML size change			
		
	Univariate (95% CI)	*P*	Multivariable (95% CI)†	*P*	Univariate (95% CI)	*P*	Multivariable (95% CI)†	*P*
*No ROA*								
Walking on a flat surface	**0.15 (0.05, 0.26)**	**0.005**	**0.14 (0.06, 0.22)**	**0.001**	**0.30 (0.05, 0.54)**	**0.018**	**0.29 (0.11, 0.48)**	**0.002**
Going up and down stairs	**0.16 (0.04, 0.28)**	**0.008**	**0.15 (0.05, 0.25)**	**0.003**	**0.33 (0.05, 0.60)**	**0.020**	**0.33 (0.09, 0.57)**	**0.006**
At night while in bed	**0.13 (0.03, 0.23)**	**0.014**	**0.11 (0.04, 0.18)**	**0.002**	0.22 (-0.01, 0.45)	0.066	**0.21 (0.04, 0.38)**	**0.014**
Sitting or lying	0.06 (-0.03, 0.15)	0.172	0.07 (-0.01, 0.15)	0.073	0.11 (-0.10, 0.31)	0.314	0.14 (-0.04, 0.33)	0.130
Standing upright	0.06 (-0.03, 0.15)	0.160	0.08 (-0.001, 0.16)	0.054	0.12 (-0.09, 0.33)	0.271	0.16 (-0.03, 0.35)	0.089
								
*ROA present*								
Walking on a flat surface	-0.02 (0.08, 0.05)	0.652	-0.02 (0.08, 0.05)	0.617	-0.02 (-0.15, 0.10)	0.686	-0.03 (-0.14, 0.09)	0.645
Going up and down stairs	0.02 (-0.07, 0.12)	0.660	0.02 (-0.06, 0.11)	0.601	0.03 (-0.14, 0.20)	0.722	0.03 (-0.12, 0.19)	0.673
At night while in bed	0.03 (-0.07, 0.14)	0.534	0.01 (-0.07, 0.09)	0.871	0.05 (-0.14, 0.25)	0.589	0.01 (-0.13, 0.16)	0.850
Sitting or lying	0.01 (-0.08, 0.10)	0.863	-0.01 (-0.08, 0.06)	0.827	0.01 (-0.15, 0.17)	0.895	-0.01 (-0.14, 0.12)	0.847
Standing upright	0.03 (-0.05, 0.12)	0.431	0.01 (-0.06, 0.07)	0.800	0.05 (-0.10, 0.21)	0.504	0.01 (-0.11, 0.14)	0.814

*Values are the change in pain sub-scale score per one standard deviation change in BML size (mm^2^), stratified by ROA.

β, beta coefficients; 95% CI, 95% confidence interval; BML, bone marrow lesion; ROA, radiographic osteoarthritis; WOMAC, Western Ontario and McMaster Universities Osteoarthritis Index.

**Medial tibial, medial femoral, lateral tibial, and lateral femoral.

† Adjusted for age, sex, body mass index, leg strength, quality of life, and baseline pain sub-scale score depending on the model. Boldface denotes statistically significant result.

Additional analyses in which we adjusted for baseline pain medication use or changes in pain medication did not alter our results, nor did separate adjustments for nonsteroidal anti-inflammatory drugs (NSAIDs). The results also remained unchanged after adjustment for tibial bone area and subchondral bone mineral density (sBMD).

### Knee replacement surgery

There were 12 total knee replacements from baseline to the five-year follow-up and baseline BML data assessed using the ordinal scale were available on all of these. The ordinal and areal BML measures were done by two separate readers and the correlation between the two was high (r = 0.79, *P *< 0.001).

Seventy-five percent (9/12) of participants who had a knee replacement had a BML at baseline. Table 5 examines the relationship between knee replacement surgery and baseline BMLs. An exploratory analysis revealed that in univariate analysis baseline BMLs in the right knee predicted incident knee replacement of the left, right, and right and left knee combined. Baseline BML severity of the right knee was a stronger predictor of a right knee replacement (OR 2.75/unit, *P *< 0.01); however, also predicted left knee replacement (OR 1.92/unit, *P *< 0.01).

**Table 5:** Relationship between knee replacement surgerynd baseline BMLs of the right knee*

	Univariate analysis		Multivariate analysis	
		
	OR (95% CI)	*P-*value	OR (95% CI) †	*P-*value
*Left knee replacement (n = 7)*				
BML severity (0 to 8)	**1.92 (1.40, 2.62)**	**<0.01**	**2.78 (1.58, 4.90)**	**<0.01**†
BML presence/absence	4.60 (0.88, 24.05)	0.07	**12.85 (1.82, 90.91)**	**0.011**†
*Right knee replacement (n = 8)*				
BML severity (0 to 8)	**2.75 (1.81, 4.18)**	**<0.01**	**2.88 (1.84, 4.52)**	**<0.01**†
BML presence/absence #	**20.75 (3.17, α)**	**<0.01**	**22.63 (3.72, α)**	**<0.01**†
*Knee replacement right and left (n = 12)*				
BML severity (0 to 8)	**2.04 (1.55, 2.69)**	**<0.01**	**2.10 (1.13, 3.90)**	**0.019**‡
BML presence/absence	**5.67 (1.51, 21.32)**	**0.01**	5.67 (0.62, 51.77)	0.124‡

*No knee replacement versus a knee replacement of the left, right, and right and left combined and baseline BMLs (measured on the ordinal scale and ranged from 0 to 12, which was the sum of the BML scores at all four sites).

95% CI, 95% confidence interval; BMLs, bone marrow lesions; OR, odds ratio

#Using exact logistic regression because all 8 subjects who had a right knee replacement had a BML present.

†Adjusted for age and sex.

‡Further adjusted for body mass index, knee pain, leg strength, cartilage defects, tibial bone area, and radiographic osteoarthritis.

Boldface denotes statistically significant result.

In multivariable analysis, BML presence and severity predicted right and left knee replacement after adjustment for age and sex. A further adjusted model examining knee replacements of the right and left knee combined revealed that BML severity predicted knee replacement after adjustment for a large number of confounders (OR 2.10, *P *= 0.019). A consistent trend to an association was observed for presence of any BML at baseline and knee replacement surgery of the right and left combined, but this did not achieve statistical significance in the adjusted model (OR 5.67, *P *= 0.124), although the OR did not change from the univariate analysis.

## Discussion

This longitudinal study describes the natural history and clinical significance of BMLs in a randomly selected population of older adults. While incidence rates were low, BMLs (assessed by measuring maximal area) were not static, with around half either worsening or improving over the study time-frame. Change in BML size was associated with changes in pain, but only in those without established ROA. In an exploratory analysis we also found that baseline BML severity independently predicted knee joint replacement surgery.

This is the first study to report the natural history of BMLs in a community based sample. Many of the previous studies have been performed in symptomatic OA cohorts, or in asymptomatic cohorts, which are not generalizable to the older population. We found that 43% exhibited one or more BMLs at baseline. In those with ROA the prevalence was similar. This is lower than in studies of patients with symptomatic OA (57 to 82% [6,14,18,19]). In the whole population, of the BMLs present at baseline, 49% showed a change in size, with similar proportions both worsening (24%) and improving (25%). Davies-Tuck *et al. *concluded that in a healthy, pain-free population BMLs develop at a slower rate than has been reported in OA populations, and that BMLs are more likely to resolve [17]. Similarly we found that BMLs increase at a slower rate in those without ROA. However, there were no significant differences in the rate of decreasing/resolving BMLs between the two subgroups. We found that 8% and 14% of BMLs completely resolved in those with and without ROA, respectively. This is much lower than Davies-Tuck *et al.*'s study in healthy asymptomatic subjects which reported that 46% resolved [17]. In subjects with prevalent knee OA or at risk for OA, Roemer *et al. *reported that nearly 41% resolved [15]. The conflicting data on the natural history of BMLs may be due to a combination of factors; including different BML grading systems among studies, the diversity within study samples, as well as the variation in study designs. We assessed BMLs by measuring the maximal area at baseline and follow-up. We then calculated whether there was an actual change in BML size from baseline to follow-up using the LSC [36], which takes into account measurement error and the correlation between measurements at baseline and follow-up. This formula provides a realistic and clinically relevant tool to identify detectable difference greater than that expected from measurement error.

In our study, the incidence of new BMLs in subjects who were BML free at baseline was low (7%). Most studies which report BML incidence have been performed in symptomatic OA cohorts [6,14,37]. Hunter *et al. *reported that a new BML developed in 20% of knees in a population with primary knee OA [6]. Similarly, Kornaat *et al. *reported that BML incidence was 21% in patients with OA [14]. In subjects with prevalent knee OA or at risk for OA, Roemer *et al. *reported an incidence of nearly 33% [15]. Our incidence rate of 7% is lower than that being described in symptomatic populations; in fact, it is even lower to what was reported in asymptomatic subjects without clinical knee OA (14%) [17]. One reason could be because this was a community based sample. However, as we have seen, the natural history of BMLs in similar populations is quite variable. It is likely that multiple factors contribute to the development of BMLs. A recent study has demonstrated a possible influence of physical effects on BMLs. In a cross-sectional design, Stehling *et al. *found that the prevalence of bone marrow abnormalities increased with the level of physical activity [38]. Our current study is the first to report BML incidence in a community based sample and it may be that BMLs vary considerably in nature because they are a result of many contributing factors.

We have found the relationship between BMLs and a change in knee pain is different for those with and without ROA. A change in BML size was associated with changes in pain as assessed by WOMAC scores, only in those without ROA, even after adjustment for a large number of factors that have been linked to knee pain [39]. A one-unit change in pain score would require a 140 mm^2 ^increase or decrease in BML area. This novel finding suggests that fluctuating knee pain may be attributable to BMLs in those participants with early stage disease. One explanation could be that once the disease progresses, there is other structural pathology contributing to knee pain. Indeed, we did find that those with ROA were more likely to have a BML present at multiple sites, so perhaps a change in one BML may not result in a symptomatic change because of other BMLs present. To support this, a previous cross-sectional study from this cohort using the ordinal BML scores found that prevalence of knee pain increased with the number of sites BMLs were present on [26]. This was independent of knee ROA. Other studies have also suggested that the size of BML is strongly related to knee pain [3,16,40]. These studies included people with ROA; therefore, it is unclear why, in the current study, BML changes were not associated with pain changes in those with ROA. This finding will need to be confirmed in future studies.

Further analysis with the WOMAC pain sub-scales demonstrated consistent results in those without ROA. We found that changes in BML size was associated with changes in knee pain when walking on a flat surface, going up and down stairs, and at night while in bed.

Interestingly, to the best of our knowledge, this is the first study to demonstrate that a decrease in BML size was associated with an improvement in knee pain. This relationship was seen in those without ROA. There are increasing data to suggest that BMLs are reversible [14,15,17] and using areal measure of BML size, we have found that a decrease is associated with a positive clinical outcome. This has important implications for intervention studies. Currently there is no disease-modifying osteoarthritis drugs (DMOADs) available to modify structural progression in OA; therefore, structure modification is now a primary aim in clinical drug trials. We believe there is increasing evidence to suggest that BMLs are a promising target. BMLs predict important disease outcomes such as cartilage loss and knee replacement, have the potential to regress and resolve, and are strongly linked to knee pain. Therefore, by targeting BMLs, it may be possible to slow disease progression as well as reduce pain in patients with OA. BMLs are visualised using standard fluid-sensitive sequences; however, new advanced imaging analysis techniques (such as T1rho and T2 relaxation time quantification, and delayed gadolinium-enhanced MRI of cartilage (dGEMRIC)) have been developed. dGEMRIC measures glycosaminoglycan (GAG) concentrations in articular cartilage and GAG content can change quickly, therefore dGEMRIC can be used to determine if altering BML natural history improves cartilage biochemistry. There is no doubt that both standard and advanced MRI techniques will play an important role in guiding future treatments in OA.

Our exploratory analysis of the relationship between BMLs and knee replacement surgery revealed that baseline BML severity independently predicted knee replacement both before and after adjustment for confounding factors. The estimate changed little after adjustment for pain severity and radiographic change, indicating that the effect of BMLs on joint replacement was not mediated through these well-established drivers of joint replacement [20,21]. This suggests that BMLs may themselves be a marker of fast progression and this in turn could explain why BMLs of the right knee predicted both right and left knee replacements. However, in view of the small numbers of knee replacement, these results need to be interpreted with caution and require confirmation in larger studies.

We have previously published data on the demographic associations with BMLs. In a separate study we showed that BMLs were more common with increasing age, male sex, and increasing BMI [41]. In the current study we did not find an association between age and BMLs; however, male sex remained a predictor of BML increases. It is unclear why males are more likely to develop BMLs. It is possible this is linked to knee trauma and knee injuries but systemic and metabolic factors may also play a role. Identifying risk factors and biomarkers for disease outcomes such as BMLs is important as it might shed light on the pathogenesis of OA. It is now understood that BMLs are an important feature in OA; however, further work is required to identify a more complete set of risk factors which should include both demographic, environment, and lifestyle factors, combined with MRI biomarkers.

This study does have potential limitations. First, for the current study, 704 were not included due to decommissioning of the MRI scanner at follow-up. There were no significant differences between those studied and the rest of the cohort in regards to demographics, ROA, WOMAC function or stiffness scores, AQoL scores, or leg strength. However, those studied had a modestly lower WOMAC pain score. Second, as previously mentioned, the numbers of knee replacement were limited. While we were able to adjust for many confounding factors in the knee replacement analysis, we had insufficient data on tibial cartilage loss to adjust for this, as we only had follow-up cartilage volume data on 3 out of the 12 subjects who underwent knee replacement surgery. We also did not have data on effusion which has been shown to predict knee replacement [22]. Last, BML area was measured from the slice with the greatest BML size. This may bias towards shallow but flat lesions; however, it is customary to measure BMLs this way. The majority of previous studies also grade BMLs on the slice with the greatest BML size; however, they use a semi-quantitative scale (0 to 3) rather than an areal measure. We acknowledge that our measure of BMLs is only a surrogate measure of volume. Recent methods have been developed to measure BML volume using a autoregression model, as well as BML signal intensity [42,43]. It is our view that the slice thickness (4 mm) and interslice gap (0.5 to 1.0 mm) of our imaging protocol was too large to estimate volume with sufficient accuracy. Both the slice thickness and the interslice gap are likely to impact on the areal BML measurements. It is possible that a shallow BML may not be detected if it lies within the interslice gap. Also, some lesions may be underestimated depending on where the slice has been taken. Smaller slice thicknesses allow for more slices to be taken and thus help to reduce measurement error. Although we acknowledge these factors as limitations it is important to consider the effect sizes we have shown. A 140 mm^2 ^change in BML areal size led to a one-unit change in pain. This change is greater than what would be expected due to measurement error alone, as our calculated LSC was 25 mm^2^.

## Conclusions

In conclusion, BMLs (assessed by measuring maximal area) were not static, with similar proportions both worsening and improving in this population-based sample. A change in BML size was associated with changes in pain in those without ROA. This finding suggests that fluctuating knee pain may be attributable to BMLs in those participants with early stage disease. Baseline BMLs also predicted knee replacement surgery. These findings suggest therapeutic interventions aimed at altering the natural history of BMLs should be considered.

## Abbreviations

AQoL: Assessment of Quality of Life; BMI: body mass index; BMLs: bone marrow lesions; CI: confidence interval; CV: coefficient of variation; dGEMRIC: delayed gadolinium-enhanced MRI of cartilage; GAG: glycosaminoglycan; ICC: intraclass correlation coefficient; JSN: joint space narrowing; LSC: least significant criterion; MRI: magnetic resonance imaging; NSAIDs: nonsteroidal anti-inflammatory drugs; OA: osteoarthritis; ROA: radiographic osteoarthritis; SD: standard deviation; TASOAC: Tasmanian Older Adult Cohort; TKR: total knee replacement; OR: odds ratio; WOMAC: Western Ontario and McMaster Universities Osteoarthritis.

## Competing interests

The authors declare that they have no competing interests.

## Authors' contributions

DD carried out analysis and interpretation of data, and prepared the manuscript. SQ participated in analysis and interpretation of the data, and critically revised the manuscript. CD designed and carried out the study planning, carried out data collection, participated in interpretation of data, and critically revised the manuscript. TW participated in interpretation of the data, and critically revised the manuscript. GZ carried out data collection, participated in interpretation of the data, and critically revised the manuscript. FC designed and carried out the study planning and critically revised the manuscript. GJ designed and carried out the study planning, participated in analysis and interpretation of the data, and critically revised the manuscript. All authors have read and approved the final manuscript.
